# Disturbed sleep and patterns of psychiatric symptoms and function in a school-based sample of adolescents

**DOI:** 10.1177/13591045221125479

**Published:** 2022-09-27

**Authors:** Lie Åslund, Anna Andreasson, Mats Lekander, Eva Henje, Inga Dennhag

**Affiliations:** 1 Department of Clinical Neuroscience, 27106 Karolinska Institutet, Sweden; 2 Centre for Psychiatry Research, Stockholm Health Care Services, Region Stockholm, CAP Research Centre, Sweden; 3 Stress Research Institute, 123910 Stockholm University, Sweden; 4 Department of Clinical Science, 59588 Umeå University, Sweden

**Keywords:** adolescent, sleep problems, psychiatric disorders, comorbidity, functional disability, PROMIS, physical activity, peer relationship, family relationship

## Abstract

**Background:**

Sleep problems are common in adolescence and often related to psychopathology and impaired functioning. However, most studies have used summative scores, and little is known about how adolescents with disrupted sleep perceive their specific symptoms and dysfunctions. This study explored differences in levels of psychiatric symptoms and functional ability between Swedish adolescents with and without self-reported disturbed sleep in a school-based sample.

**Methods:**

Swedish adolescents (*n* = 618, mean age 15.7+/-1.9yrs) answered the PROMIS pediatric measures for fatigue, anxiety, depression, pain interference, anger, physical activity and peer and family relationships. Logistic regression analyses were performed to assess differences between respondents with and without disturbed sleep.

**Results:**

Disturbed sleep was associated with higher levels of symptoms of fatigue, anxiety, depression, anger and pain interference, as well as lower functional abilities in terms of physical activity and peer- and family relationships. Adolescents reporting disturbed sleep generally displayed a pattern of impaired executive functioning, internal emotional distress and school- and sleep related worry and dysfunction, as compared to physical disability, aggressive behavior, stress and generalized worry.

**Conclusions:**

The present study adds to the understanding of how disturbed sleep and specific psychiatric symptoms and functional ability are interrelated, which may also have clinical implications.

## Introduction

Disordered sleep, especially insomnia, is common in adolescence (Dohnt et al., 2012). An individual with insomnia displays difficulties in falling asleep, in staying asleep as long as desired, and/or suffers from early awakenings for at least three nights per week over at least 3 months, with associated clinically significant functional impairment (American Psychiatric Association, 2013). Diagnostic and statistical manual of mental disorders (fifth ed.). Washington). Adolescent insomnia has a mean age of onset of 11 years with difficulties initiating sleep as the most common symptom (Johnson et al., 2006). 10 % of adolescents in the general population meet criteria for insomnia and one third of them report “at least some” insomnia symptoms (Dohnt et al., 2012). Sleep loss can affect mood states in this age group, with symptoms of depression and anger –in addition to fatigue– being higher after disturbed sleep (Short & Louca, 2015). When resulting in sleepiness, disordered sleep in adolescents also negatively affects executive functioning (Anderson et al., 2009) and contributes to increased irritability, inattention, and lack of motivation (Carskadon, 2011). In addition, experimental sleep restriction results not only in self-reported negative affective functioning but also in negative affective behavior in social peer contexts (McMakin et al., 2016), altogether suggesting that disturbed sleep may cast shadows not only on emotional functioning but also on academic performance and functioning.

The transition from childhood to adolescence seems to present an increased risk of developing both sleeping problems and comorbid psychiatric symptomatology (McMakin & Alfano, 2015). In fact, more than half of adolescents with insomnia fulfill criteria for another comorbid psychiatric disorder (Johnson et al., 2006) most commonly Major Depressive Disorder (MDD) or anxiety disorders (Johnson et al., 2000). Sixty-six percent of adolescents with insomnia also presented depressive symptoms (Amaral et al., 2017) in one study focusing on sleep and MDD. As many as 90% of children with anxiety disorders in late childhood/early adolescence also report symptoms of disordered sleep (Chase & Pincus, 2011). In addition to psychiatric problems, disordered sleep often occurs together with pain in both adults (Kundermann et al., 2004) and adolescents (Kanstrup et al., 2014), and experimental sleep deprivation has been shown to contribute to increased pain sensitivity (Haack et al., 2009; Kundermann et al., 2004). In adolescents with chronic pain, 60% suffer from insomnia and the link between pain, depression and functional disability was mediated by insomnia (Kanstrup et al., 2014). Thus, insomnia is common in adolescents and is connected with other problems that are likely to both contribute to sleep problems and to be negative consequences of disordered sleep. Furthermore, for adolescents with disordered sleep and co-morbid anxiety disorders and/or depression a priority in targeting disordered sleep may decrease symptoms of the comorbid states (Aslund et al., 2018; Meltzer & Mindell, 2014) (Åslund et al., 2020) as well as fatigue and anger (Van Dyk et al., 2017).

Self-reported symptomatology is generally restricted to the use of inventories and their summative scores, indicating a general degree of symptoms or absence or presence of a specific disorder. Not much is thus known about specific self-reported symptoms or functional disabilities that distinguish adolescents with disordered sleep from those without sleep problems. The aim of the present study was therefore to explore and elucidate in greater detail the variety of self-reported symptoms and functional abilities between adolescents with and without self-reported disturbed in a sample of Swedish adolescents, and to identify what specific symptoms and functional abilities may differentiate these groups.

## Methods

### Data collection and procedure

Data was collected from four schools of different socioeconomic standards in a medium-sized town and its surroundings in the northern part of Sweden during school year 2018–2019. The purpose of that project was to develop new instruments for screening and treating mental illness among young patients. Ethical approval was given by the Regional Ethical Review Board in Umeå, Sweden (number 2018/59-31). Verbal and written information was given to students by their teachers and written consent was obtained from those who chose to participate. From students <15 years, informed consent was obtained by the parents/guardians. Participants received a code to access the web survey during school hours, with assistance from a teacher and research assistant if required. Due to all questions having to be answered to finish the survey, no missing data was created. Participants were provided with a snack while filling out the survey and received a cinema gift card upon completion.

The study population was a convenience sample with students aged 12-20. A total of 897 students were asked to participate in the study and 618 (69%) agreed to do so.

### Outcome measures

#### Disturbed sleep

Respondents were defined as having “disturbed sleep” if they responded “often” or “always” (as opposed to “*never*” or “*sometimes*”) to at least one of two sleep-related items from the larger data collection (described in 2.1): “*I have trouble sleeping”* (from the Revised Children’s Anxiety and Depression Scale (Chorpita et al., 2000)) or “*I sleep poorly” *(from Beck Youth Inventory II (Beck, 2001)) while non-disturbed sleepers responded “never” or sometimes” to these items. A dichotomous variable called “Disturbed sleep” was created.

### Symptoms and functions

All respondents completed the Patient Reported Outcomes Measurement Information System (PROMIS) pediatric measures of fatigue (Lai et al., 2013; Quinn et al., 2014), anxiety (Quinn et al., 2014), depressive symptoms (Quinn et al., 2014), pain interference (Quinn et al., 2014; Varni et al., 2014), anger (Quinn et al., 2014), physical activity (Tucker et al., 2014), peer relationships (Dewalt et al., 2013) and family relationships (Bevans et al., 2017). PROMIS were developed using qualitative and item response theory methods to measure physical, mental and social health (Cella et al., 2010). PROMIS pediatric measures documents self-reported symptoms and functioning over the previous 7 days with a 5-option response with higher scores indicating more of the measured symptom or function. PROMIS measures are constructed to allow items to be analyzed separately, permitting a more flexible data collection. Swedish translations of the PROMIS pediatric measures have been established (Chaplin et al., in manuscript). Definition of the PROMIS measure domains are found at www.healthmeasures.net. For detailed information on the measures used in the data collection (version, number of items), see Supplementary Table 1.

### Statistical analyses

All statistical analyses were performed using Stata, version 16.0. To characterize the sample and the subgroups of respondents with and without disturbed sleep, independent sample *t-*tests were used on demographic variables. Due to the dependent variable “Disturbed sleep” being dichotomous, logistic regressions were performed to analyze differences in levels of symptoms and functions between the two groups. Analyzes were performed on summative PROMIS-scores, as well as on individual item-scores, with  *p*< 0.05 considered statistically significant. All effect sizes were calculated as standardized Cohen’s *d’s* (Cohen, 1992) with 95% confidence intervals (95% CIs). Effect sizes were considered “small” if *d* =  0.0–0.4, “moderate” if *d*  =  0.4–0.7 and “large” *d*  ≥  0.7 (Cohen, 1988; Matthey, 1998). As the study had an explorative approach, all results were analyzed and no limitations for possible Type-I errors were made.

## Results

### Sample characteristics

#### Disturbed sleep

Almost one quarter of respondents reported enough sleep problems to be classified as having “disturbed sleep” (Table 1).

**Table 1.** Demographic data of all respondents and subgroups of respondents reporting “disturbed sleep” or “non-disturbed sleep”.

	All	Disturbed sleep	Non- disturbed sleep	*p*-value
*n* (%)	618 (100)	142 (23)	476 (77)	
Age, mean (SD) years	15.7 (1.9)	16.0 (1.8)	15.6 (1.9)	0.029
Female, *n* (%)	381 (61.7)	88 (61.9)	293 (61.6)	0.96
Born in Sweden, *n* (%)	563 (90.8)	126 (88.7)	437 (91.4)	0.18
Parent/Guardian working, *n* (%)				
Parent 1	534 (86.1)	113 (79.6)	421 (88.1)	0.002
Parent 2	538 (86.8)	119 (83.8)	419 (87.7)	
Contact with care provider (12 months)				
*Psychiatric problems*				
Primary care, *n* (%)	29 (6.0)	13 (12.4)	16 (4.2)	0.016
Youth health center, *n* (%)	23 (4.7)	8 (7.6)	15 (3.9)	
Specialist care, *n* (%)	15 (3.1)	4 (3.8)	11 (2.9)	
*Physical problems*				0.71
Primary care, *n* (%)	84 (14.0)	20 (14.5)	64 (13.9)	
Youth health center, *n* (%)	12 (2.0)	8 (1.7)	8 (1.7)	
Specialist care, *n* (%)	9 (1.5)	7 (1.5)	7 (1.5)	
School, successful in all subjects (%)	532 (85.8)	109 (76.8)	423 (88.5)	<0.001

Note. Independent sample *t*-tests were used to analyze demographic data.

#### Demographics

Table 1 also presents demographic data for the total sample and for the two respective subgroups (disturbed sleep; non-disturbed sleep) separately. A majority of respondents were female, born in Sweden and with both parents/guardians working. A minority of respondents reported having been in contact with a primary care unit, a youth health center or a specialist care unit for psychiatric and/or physical problems during the last 12 months.

### Differences in levels of symptoms and functions between poor and good sleepers

There was a statistically significant difference between the groups for summative scores for all measure domains (*p*≤0.001), with respondents with disturbed sleep reporting higher scores for symptom domains and lower scores for functional domains (Table 2). Coefficients for group differences ranged from 0.24 to 1.21, with small to large effect sizes. Among the five symptom domains, fatigue differed most between the groups, followed by anxiety, depressive symptoms, pain interference and anger (coefficients = 0.74–1.21; *d’s =* 0.53–0.93). The functional domains, i.e., physical activity, peer relationships and family relationships, also differed significantly between the groups, but relatively less as compared to symptom domains (coefficients = −0.56-0.24; *d’s* = 0.22–0.44). The difference was in the moderate or high range for five measure domains (fatigue, anxiety, depression, pain interference and anger).

**Table 2.** Results of the differences between the groups (disturbed sleep; non-disturbed sleep) on summative scores for all measure domains.

	Poor sleepers		Good sleepers						
PROMIS® measure domain	M	SD	M	SD	Coef	95% Conf. Interval		*p*-value	Effect size Cohen’s *d*
						Cl-	Cl+		
Fatigue	67.47	21.65	46.17	18.70	1.21	0.96	1.46	<0.001	0.93
Anxiety	28.58	12.10	22.44	8.46	0.90	0.66	1.18	<0.001	0.64
Depressive symptoms	32.39	14.30	24.56	10.32	0.74	0.52	0.95	<0.001	0.66
Pain interference	34.07	16.40	26.69	11.53	0.74	0.48	1.00	<0.001	0.53
Anger	20.28	9.74	15.34	6.76	0.67	0.66	0.88	<0.001	0.61
Physical activity	31.35	9.58	29.49	8.74	−0.24	−0.45	−0.03	0.024	0.22
Family relationships	16.49	7.77	13.45	6.70	−0.44	−0.64	−0.24	<0.001	0.44
Peer relationships	33.25	10.17	29.03	10.49	−0.56	−0.82	−0.30	<0.001	0.43

Note. Logistic regressions were used to calculate differences between the groups (disturbed sleep; non-disturbed sleep). Domains are sorted according to the size of the regression coefficient.

### Differences in specific symptoms and functions between respondents with disturbed sleep and non-disturbed sleep

Table 3 and 4 shows the difference between the groups for the mean score of each of the *n* = 116 items, sorted according to the size of the regression coefficient for each domain. All differences were significant between the groups (*p* ≤ 0.001) with the respondents with disturbed sleep reporting higher scores for the symptom domains and lower scores for the function domains for all items, with two exceptions. Regression coefficients ranged from 0.00-0.90 and with small 0.02 to large (0.91) effect sizes. For the 20% (*n* = 23) items with the largest regression coefficient (regardless of domain), the majority of the items (74%) originated from the scale of fatigue (17 items), followed by pain interference (3 items), anxiety (2 items) and anger (1 item). Regression coefficients ranged from 0.58-0.82 and effect sizes ranged from small (0.48) to large (0.91). For the 20% (*n* = 23) items with the smallest regression coefficient (regardless of domain), majority of the items (39%) originated from the scale of physical activity (9 items), followed by peer relationships (5 items), anxiety (4 items), pain interference (3 items) and family relationships (2 items). For more details on regression coefficients, confidence intervals, (95% CI’s), *p*-values and effect sizes, see Supplementary Table 2.

**Table 3.** Differences in mean item score, as displayed by the regression coefficient, between respondents in the two groups (disturbed sleep; non-disturbed sleep) for all *n* = 116 items.

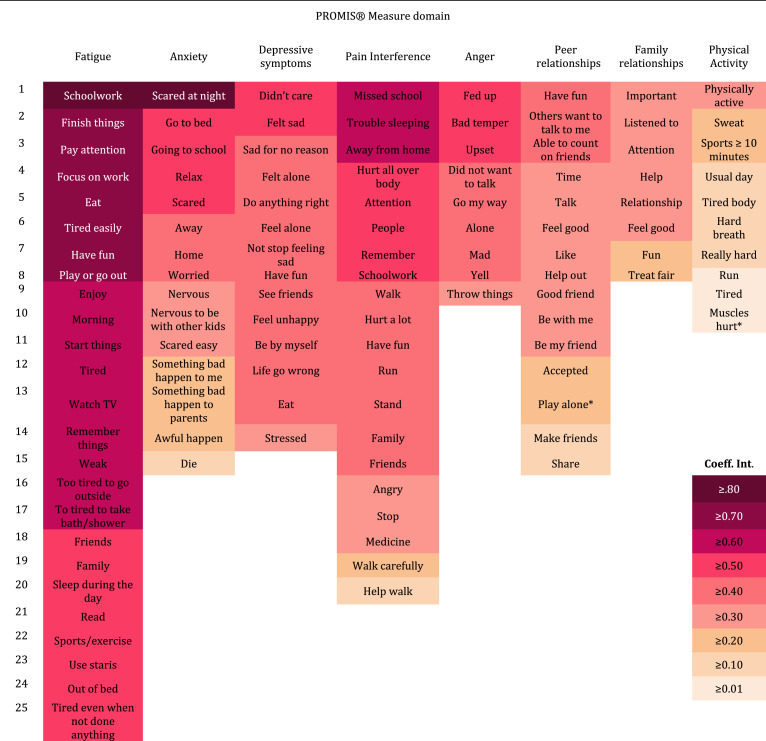

Note. PROMIS domain are sorted according to the difference (size of the regression coefficient, see Table 2) between those who reported disturbed sleep and those who did not. PROMIS items are sorted by the size of the regression coefficient for each domain separately and are referred to by a description to indicate the content of the item and item number. For example, “Schoolwork” in the fatigue domain refers to the question “*Being tired made it hard for med to keep up with my schoolwork”* with the color code indicating the size of the regression coefficient (difference between respondents reporting disturbed sleep vs. non-disturbed sleep, darker color indicating higher value). See Supplement 1 for detailed statistics. The number of items per category of regression coefficient were as follows as follows: two items≥0.8; seven items≥0.7; 12 items ≥0.6; 22 items≥0.5; 29 items≥0.4; 23 items≥0.3; 10 items≥0.2; eight items≥0.1; three items<0.01.* indicates that respondents with disturbed sleep reported less symptoms/higher function than those who reported non-disturbed sleep.

**Table 4.** Full item-questions for the items with the largest and smallest regression coefficient (differences between respondents reporting disturbed sleep vs. non-disturbed sleep).

PROMIS® measure domain	Items with largest regression coefficient	Items with smallest regression coefficient
Fatigue	“Being tired made it hard for me to keep up with my schoolwork”	“I felt tired even when I had not done anything”
	“I had trouble finishing things because I was too tired”	“It was hard for me to get out of bed in the morning because I was too tired”
	“I was so tired it was hard for me to pay attention”	“I was too tired to go up and down a lot of stairs
Anxiety	“I woke up at night scared”	“I was worried I might die”
	“ I worried when I went to bed at night”	“I felt like something awful might happen”
	“I was afraid of going to school”	“I worried that something might happen to my parents or guardians”
Depressive symptoms	“I didn’t care about anything”	“I felt stressed”
	“I felt sad”	“I felt too sad to eat”
	“I felt sad for no reason”	“I felt everything in my life went wrong”
Pain interference	“I missed school when I had pain”	“I needed help walking when I was in pain”
	“I had trouble sleeping when I had pain”	“I walked carefully when I was in pain”
	“It was hard for me to be away from home because I had pain”	“My pain was so bad that I needed to take medicine to treat it”
Anger	“I felt fed up”	“I was so angry I felt like throwing something”
	“I had a bad temper”	“I was so angry I felt like yelling at somebody”
	“I felt upset”	“I felt mad”

Note. The *n* = 5 domains with effect size >0.5 of the difference on summative scores for measure domains.

## Discussion

This study examined differences in levels of symptoms and functional domains between adolescents with self-reported disturbed sleep and non-disturbed sleep in a school-based Swedish cohort. A more detailed examination of differences in specific symptomatology and functional abilities between these groups was also performed. When comparing summative scores for the two groups, the differences were significant for all measures, with the respondents with disturbed sleep being worse off. This group thus reported higher symptom levels overall, with fatigue, anxiety, depression, anger and pain interference differing the most as compared to those reporting non-disturbed sleep. Effect sizes were moderate to large. For the functional domains (physical activity, peer- and family relationships), respondents with disturbed sleep reported lower functioning, but these domains differed relatively less between the groups (with small to medium effect sizes). These findings are consistent with previous observations in adolescents in which sleep disturbances have notably been associated with depression and anxiety (Short & Louca, 2015; Van Dyk et al., 2017).

Regarding specific psychiatric symptoms and functional abilities, the respondents with disordered sleep reported higher scores for all items in the symptom domains and lower scores in the function domains (with two exceptions out of all 116 items). For the 20% items with the largest regression coefficient, all items originated from a symptom-domain rather than a functional domain. Three quarters of the items originated from the fatigue scale. The last observation is not surprising considering the close relation between sleep and recovery (Lo et al., 2016), even though it should be noted that motivational factors (which are also related to sleep (Axelsson et al., 2020)) are also highly related to fatigue, for example to muster energy in order to perform an activity. The rest of these items originated from the domains of pain interference, anxiety and anger. This suggests that sleep disturbances are more strongly associated with these symptoms, as compared to depressive symptoms or physical/social function. For the 20% items with the smallest regression coefficient, 70% of items were related to functional domains, and more specifically to physical activity, suggesting that these functions are not specifically impaired due to disturbed sleep.

The items with largest and smallest regression coefficients were further examined for the five most relevant measure domains (with d>0.5 of the summative score difference between respondents reporting disturbed vs. non-disturbed sleep). For fatigue, the groups differed most regarding executive functioning (e.g., attention, finishing tasks) and least in impaired physical function (e.g., get out of bed, taking the stairs). This is in line with previous reports on disturbed sleep, attention and working memory in adolescence (Lo et al., 2016). For depression, poor sleepers showed more internal emotional distress (e.g., feeling of sadness) while the least difference regarded general stress and loss of feeling of control. Even though a relation between sleep problems and stress has been documented in adolescence (Astill et al., 2013; Mesquita & Reimão, 2010), the results of the present study suggest that poor sleep could be less related to stress than more internal depressive symptoms.

Importantly, regarding anxiety symptoms, worrying at bedtime and feeling scared when in school were more related to disordered sleep as compared to generalized anxiety. This may imply that insomnia-specific rumination, which is known to be related to hyperarousal and insomnia in adults (Palagini et al., 2017), is applicable for adolescents too. These results support the notion that insomnia treatment targeting anxiety-driven rumination at bedtime can improve both the anxiety and the disordered sleeping patterns in adolescence (Åslund et al., 2020). Comorbidity between disordered sleep and anxiety in general could benefit from more detailed examination in future studies, both when examining the prevalence of these disorders, and with regard to sleep intervention outcomes. Respondents with disordered sleep scored higher on self-reported anger and tended to internalize anger (e.g., feeling upset or fed up) to a greater extent compared to those with normal sleep., while more overt aggressive behaviors (e.g., feeling like yelling/throwing) differed less between the groups. These results suggest a relationship between disordered sleep and “quiet” anger, rather than aggressive behavior, much like the results for the depressive symptoms domain.

For pain interference, the groups differed most with regard to school attendance and sleep quality, as opposed to physical exercise and medication. This suggests that although both groups might use medication to treat their pain to an almost equal extent, adolescents reporting disturbed sleep have a reduced school attendance due to pain when compared to their peers who do not report disturbed sleep. These results are consonant with insomnia being a mediator of the relationship between pain and functional disability in adolescents (Kanstrup et al., 2014).

There are several strengths and limitations of the present study. The observational and cross-sectional design precludes any conclusions regarding causality in the observed relationships between disturbed sleep on the one hand and symptomatology and functional ability on the other. In future studies, it would be beneficial to use repeated measures over time in order to achieve a more solid understanding of how changes in sleep problems might aggravate symptoms of psychopathology and impact overall function, and vice versa. It should be noted that respondents were not geographically stratified and did not fully match the Swedish pediatric population (e.g., the gender ratio), making the generalizability of the results uncertain. However, the sample is large and heterogeneous regarding socio-economic status due to the data being collected in several schools. Due to the explorative approach of the study, no restrictions were applied to data concerning Type-I error, increasing the risk of false positive findings and future studies are necessary to validate the current findings.

## Conclusion

The present study supports the observation that disturbed sleep is associated with higher levels of symptoms of fatigue, depression, anxiety, anger and pain interference, as well as lower physical activity and peer- and family relationships in adolescents. It indicates that disturbed sleep is more related to executive functioning, internal emotional distress and school- and sleep related worry and dysfunction compared to physical disability, aggressive behavior, stress and generalized worry. Long-term follow-up studies as well as assessment of interventions targeting sleep problems in adolescents to analyze mediating and moderating factors and to better understand the direction of causality are warranted. These research findings contribute to our understanding of how sleep and specific psychiatric symptoms and functional abilities are interrelated, which may have implications for the future development of improved treatment for insomnia as well as for anxiety disorders and depression in adolescents. Targeting specific components of comorbid psychiatric disorders, such as anxiety-driven rumination about sleep and internalized anger and depression, could enhance treatment effects following psychological treatments of insomnia in adolescents.

## Supplemental Material

Supplemental Material - Disturbed sleep and patterns of psychiatric symptoms and function in a school-based sample of adolescents Click here for additional data file. Supplemental Material for Disturbed sleep and patterns of psychiatric symptoms and function in a school-based sample of adolescents by Lie Åslund, Anna Andreasson, Mats Lekander, Eva Henje, Inga Dennhag Child Psychology and Psychiatry
