# Supplementary material for: Disturbed sleep and patterns of psychiatric symptoms and function in a school-based sample of adolescents
Source: Clin Child Psychol Psychiatry. 2022 Sep 27;28(4):1524–35. doi: 10.1177/13591045221125479 (PMC10540489; doi:10.1177/13591045221125479)
Supplement: Supplemental Material - Disturbed sleep and patterns of psychiatric symptoms and function in a school-based sample of adolescents [file sj-pdf-1-ccp-10.1177_13591045221125479.pdf]

Supplementary Table 1. Outcome PROMIS® Measures for pediatric self-report.

Domain	Ref.	Definition	Nb. of items	Full name
Fatigue	[21, 22]	Subjective feelings of tiredness.	25	PROMIS Pediatric Item Bank v2.0 – Fatigue
Anxiety	[22]	Fear, anxious misery, hyperarousal and related somatic symptoms.	15	PROMIS Pediatric Item Bank v2.0 – Anxiety
Depressive symptoms	[22]	Negative mood, views of self, social cognition, decreased positive affect and engagement.	14	PROMIS Pediatric Item Bank v2.0 – Depressive Symptoms
Pain interference	[22, 23]	Consequences of pain on social, cognitive, emotional, physical and recreational activities.	20	PROMIS Pediatric Item Bank v2.0 – Pain Interference
Anger	[22]	Angry mood, negative social cognitions and efforts to control anger.	9	PROMIS Pediatric Scale v.2.0 – Anger 9a
Physical activity	[24]	Capability of extremities, central regions and instrumental activities of daily living.	10	PROMIS Pediatric Item Bank v1.0 - Physical Activity
Family relationships	[26]	Being involved with the family, feeling accepted and cared for and feeling that family members, especially parents, can be trusted and depended on for help and understanding.	8	PROMIS Pediatric Item Bank v1.0 - Family Relationships - Short Form 8a
Peer relationships	[25]	Quality of relationships with fiends and other acquaintances.	15	PROMIS Pediatric Item Bank v2.0 – Peer Relationships

Supplementary Table 2. Full item question, regression coefficient (standard error) and 95% confidence intervals for differences in mean item score between respondents in the two groups (disturbed sleep; non-disturbed sleep) for all n=116 items.

	PROMIS® Measure domain							
	**Fatigue**	**Anxiety**	**Depressive symptoms**	**Pain Interference**	**Anger**	**Peer relationships**	**Family relationships**	**Physical Activity**
1	Being tired made it hard for me to keep up with my schoolwork<br><br>0.82 (0.09)<br>[0.65-0.99]	I woke up at night scared<br><br>0.80 (0.12)<br>[0.56-1.04]	I didn't care about anything<br><br>0.51 (0.09)<br>[0.34-0.68]	I missed school when I had pain<br><br>0.67 (0.13)<br>[0.41-0.93]	I felt fed up<br><br>0.59 (0.08)<br>[0.43-0.76]	I was able to have fun with my friends<br><br>-0.43(0.10)<br>[-0.63- -0.22]	I felt really important to my family<br><br>-0.39 (0.09)<br>[-0.56- -0.22]	How many days were you physically active for 10 minutes or more?<br><br>-0.30 (0.08)<br>[-0.46- -0.14]
2	I had trouble finishing things because I was too tired<br><br>0.79 (0.09)<br>[0.62-0.97]	I worried when I went to bed at night<br><br>0.58 (0.08)<br>[0.43-0.74]	I could not stop feeling sad<br><br>0.47 (0.08)<br>[0.30-0.63]	I had trouble sleeping when I had pain<br><br>0.65 (0.09)<br>[0.47-0.83]	I had a bad temper<br><br>0.51 (0.09)<br>[0.33-0.69]	Other kids wanted to talk to me<br><br>-0.40 (0.09) [-0.57- -0.22]	My parents listened to me<br><br>-0.38 (0.09)<br>[-0.56- -0.20]	How many days were you so physically active that you sweated?<br><br>-0.27 (0.09)[-0.44- -0.09]
3	I was so tired It was hard for me to pay attention<br><br>0.76 (0.09)<br>[0.58-0.93]	I was afraid of going to school<br><br>0.57 (0.12)<br>[0.34-0.81]	I felt sad for no reason<br><br>0.49 (0.08)<br>[0.33-0.65]	It was hard for me to be away from home because I had pain<br><br>0.64 (0.13)<br>[0.39-0.88]	I felt upset<br><br>0.51 (0.09)<br>[0.34-0.68]	I was able to count on my friends<br><br>-0.40 (0.09) [-0.58- -0.22]	My family paid a lot of attention to me<br><br>-0.37 (0.09)<br>[-0.55- -0.19]	How many days did you play sports for 10 minutes or more?<br><br>-0.20 (0.08)<br>[-0.36- -0.04]
4	I was so tired it was hard for me to focus on my work<br><br>0.73 (0.09)<br>[0.56-0.90]	It was hard for me to relax<br><br>0.55 (0.08)<br>[0.39-0.71]	I felt lonely<br><br>0.48 (0.08)<br>[0.33-0.64]	It hurt all over my body<br><br>0.57 (0.11)<br>[0.35-0.79]	I was so mad I did not want to talk to people<br><br>0.48 (0.09)<br>[0.30-0.66]	I spent time with my friends<br><br>-0.39 (0.09)<br>[-0.57- -0.21]	I got all the help I needed from my family<br><br>-0.34 (0.09)<br>[-0.51- -0.17]	On a <u>usual day</u>, how physically active were you?<br><br>-0.19 (0.09)<br>[-0.38- -0.01]
5	I was too tired to eat<br><br>0.72 (0.11)<br>[0.54-0.94]	I felt scared<br><br>0.51 (0.10)<br>[0.31-0.71]	I felt like I couldn't do anything right<br><br>0.40 (0.08)<br>[0.24-0.56]	It was hard for me to pay attention when I had pain<br><br>0.55 (0.09)<br>[0.38-0.72]	I was angry when things didn't go my way<br><br>0.46 (0.08)<br>[0.30-0.62]	I was able to talk about anything with my friends<br><br>-0.35 (0.08)<br>[-0.50- -0.19]	I felt I had a strong relationship with my family<br><br>-0.32 (0.09)<br>[-0.50- -0.14]	How many days did you exercise or play so hard your body got tired?<br><br>-0.16 (0.09)<br>[-0.33-0.01]
6	I got tired easily<br><br>0.72 (0.09)<br>[0.55-0.89]	I worried when I was away from home<br><br>0.47 (0.11)<br>[0.25-0.69]	I felt alone<br><br>0.49 (0.08)<br>[0.33-0.65]	It was hard to get along with other people when I had pain<br><br>0.54 (0.13)<br>[0.28-0.80]	I wanted to be alone because I was so angry<br><br>0.46 (0.09)<br>[0.29-0.64]	I felt good about my friendships<br><br>-0.35 (0.09)<br>[-0.54- -0.16]	People in my family made me feel good about myself<br><br>-0.31 (0.08)<br>[-0.47- -0.15]	How many days did you exercise so much that you breathed hard?<br><br>-0.16 (0.08)<br>[-0.33-0.01]
7	Being tired kept me from having fun<br><br>0.71 (0.10)<br>[0.53-0.90]	I worried when I was home<br><br>0.46 (0.10)<br>[0.26-0.66]	I could not stop feeling sad<br><br>0.50 (0.09)<br>[0.32-0.68]	It was hard for me to remember things when I had pain<br><br>0.52 (0.12)<br>[0.30-0.77]	I felt mad<br><br>0.46 (0.09)<br>[0.29-0.64]	I liked being around other kids my age<br><br>-0.34 (0.10)<br>[-0.53- -0.15]	My family and I had fun together<br><br>-0.29 (0.08)<br>[-0.46- -0.13]	How many days did you exercise really hard for 10 minutes or more?<br><br>-0.14 (0.08)<br>[-0.31-0.02]

PROMIS® Measure domain								
	**Fatigue**	**Anxiety**	**Depressive symptoms**	**Pain Interference**	**Anger**	**Peer relationships**	**Family relationships**	**Physical Activity**
8	Being tired made it hard for me to play or go out with my friends as much as I'd like<br><br>0.70 (0.09)<br>[0.53-0.88]	I felt worried<br><br>0.43 (0.09)<br>[0.26-0.60]	It was hard for me to have fun<br><br>0.46 (0.09)<br>[0.29-0.63]	I had trouble doing schoolwork when I had pain<br><br>0.50 (0.09)<br>[0.33-0.67]	I was so angry I felt like yelling at somebody<br><br>0.40 (0.09)<br>[0.23-0.57]	My friends and I helped each other out<br><br>-0.33 (0.09)<br>[-0.51- -0.14]	My family treated me fairly<br><br>-0.26 (0.09)<br>[-0.43- -0.09]	How many days did you run for 10 minutes or more?<br><br>-0.07 (0.08)<br>[-0.23-0.08]
9	I was too tired to enjoy the things I like to do<br><br>0.66 (0.09)<br>[0.48-0.83]	I felt nervous<br><br>0.35 (0.08)<br>[0.20-0.50]	Being sad made it hard for me to do things with my friends<br><br>0.45 (0.10)<br>[0.25-0.65]	It was hard for me to walk one block when I had pain<br><br>0.45 (0.11)<br>[0.24-0.67]	I was so angry I felt like throwing something<br>0.37 (0.09)<br>[0.20-0.54]	I was a good friend<br><br>-0.31 (0.10)<br>[-0.52- -0.11]		How many days did you exercise or play so hard that your felt tired?<br><br>-0.07 (0.09)<br>[-0.24-0.10]
10	I felt more tired than usual when I woke up in the morning<br><br>0.66 (0.08)<br>[0.50-0.81]	I felt too nervous to be with a group of kids my age<br>0.34 (0.09)<br>[0.16-0.52]	I felt unhappy<br><br>0.44 (0.08)<br>[0.27-0.60]	It hurt a lot<br><br>0.42 (0.09)<br>[0.24-0.61]		Other kids wanted to be with me<br><br>-0.31 (0.09)<br>[-0.48- -0.14]		How many days did you exercise or play so hard that your muscles burned?*<br><br>0.02 (0.09)<br>[-0.16-0.20]
11	I had trouble starting things because I was too tired<br><br>0.65 (0.08)<br>[0.50-0.81]	I got scared really easy<br><br>0.31 (0.08)<br>[0.15-0.46]	I wanted to be by myself<br><br>0.43 (0.08)<br>[0.26-0.60]	It was hard to have fun when I had pain<br><br>0.41 (0.08)<br>[0.26-0.56]		Other kids wanted to be my friend<br><br>-0.30 (0.09)<br>[-0.47- -0.13]		
12	I felt tired<br><br>0.65 (0.09)<br>[0.49-0.82]	I was worried about what could happen to me<br><br>0.26 (0.10)<br>[0.07-0.44]	I felt everything in my life went wrong<br><br>0.38 (0.08)<br>[0.22-0.54]	It was hard for me to run when I had pain<br><br>0.41 (0.08)<br>[0.26-0.56]		I felt accepted by other kids my age<br><br>-0.21 (0.09)<br>[-0.39- -0.02]		
13	I was too tired to watch television<br><br>0.64 (0.10)<br>[0.44-0.83]	I worried that something might happen to parents or guardians<br><br>0.26 (0.09)<br>[0.09-0.43]	I felt too sad to eat<br><br>0.40 (0.10)<br>[0.21-0.59]	It was hard to stay standing when I had pain<br><br>0.41 (0.10)<br>[0.22-0.60]		I played alone and kept to myself*<br><br>0.21 (0.08)<br>[0.06-0.36]		
14	Being tired made it hard for me to remember things<br><br>0.64 (0.08)<br>[0.47-0.80]	I felt like something awful might happen<br><br>0.27 (0.10)<br>[0.08-0.46]	I felt stressed<br><br>0.38 (0.08)<br>[0.21-0.51]	It was hard to do things with my family because I had pain<br><br>0.41 (0.13)<br>[0.15-0.66]		I was good at making friends<br><br>-0.18 (0.08)<br>[-0.34- -0.03]		
15	I felt weak<br><br>0.62 (0.09)<br>[0.45-0.79]	I was worried I might die<br><br>0.14 (0.12)<br>[-0.10-0.38]		It was hard to have fun with friends because I was in pain<br><br>0.40 (0.12)<br>[0.17-0.63]		I shared with other kids (food, games, pens, etc.)<br><br>-0.11 (0.09)<br>[-0.29- 0.07]		

PROMIS® Measure domain								
	**Fatigue**	**Anxiety**	**Depressive symptoms**	**Pain Interference**	**Anger**	**Peer relationships**	**Family relationships**	**Physical Activity**
16	Too tired to do things outside 0.61 (0.09) [0.44-0.79]			I felt angry when I had pain 0.37 (0.09) [0.18-0.55]				
17	I was too tired to take a bath or shower 0.61 (0.09) [0.43-0.79]			I had so much pain I had to stop what I was doing 0.34 (0.11) [0.11-0.56]				
18	I felt too tired to spend time with my friends 0.58 (0.09) [0.40-0.77]			My pain was so bad I needed medicine to treat it 0.30 (0.10) [0.10-0.50]				
19	I was too tired to go out with my family 0.55 (0.10) [0.36-0.74]			I walked carefully when I was in pain 0.27 (0.09) [0.09-0.46]				
20	I needed to sleep during the day 0.53 (0.07) [0.38-0.67]			I needed help walking when I was in pain 0.14 (0.16) [-0.17-0.45]				
21	I was too tired to read 0.53 (0.08) [0.38-0.67]							
22	I was too tired to do sport or exercise 0.52 (0.07) [0.37-0.67]							
23	I was too tired to go up and down a lot of stairs 0.51 (0.09) [0.33-0.68]							
24	It was hard for me to get out of bed in the morning because I was too tired 0.51 (0.07) [0.36-0.65]							
25	I felt tired even when I had not done anything 0.50 (0.08) [0.35-0.65]							

Note. * indicates that respondents with disturbed sleep reported less symptoms / higher function than those who reported non-disturbed sleep.
